# Post-copulatory competition in a social monogamy system: Sperm morphology correlates with components of reproductive success

**DOI:** 10.1371/journal.pone.0337846

**Published:** 2025-12-01

**Authors:** Carly E. Hawkins, Thomas P. Hahn, Jessica L. Malisch, Gail L. Patricelli

**Affiliations:** 1 Department of Ecology and Evolutionary Biology, University of California Davis, Davis, California, United States of America; 2 Department of Neurobiology, Physiology and Behavior, University of California Davis, Davis, California, United States of America; 3 Natural Reserve System, University of California Merced, Merced, California, United States of America; University of Massachusetts, UNITED STATES OF AMERICA

## Abstract

Males in socially monogamous species can achieve reproductive success through multiple tactics– by defending paternity within the social nest and siring extra-pair offspring, or both. Previous studies have found that sperm morphology may differentially affect fertilization success in extra-pair compared to within-pair matings; therefore, we explored whether sperm morphological traits can predict the probability of success within components of reproductive success. Here, we measured sperm component traits (head length and flagellum length) and derived traits (total length and flagellum:head ratio) in free-living Mountain White-crowned Sparrows (*Zonotrichia leucophrys oriantha*) and examined how these morphological traits relate to extra-pair and within-pair reproductive components of reproductive success. We found no evidence for correlations between sperm morphology and total seasonal reproductive success. However, we did find that sperm morphology appeared to be associated with whether a male was successful at acquiring extra-pair offspring or defending his own paternity within his nest: males that achieved extra-pair success had longer flagella and longer total length of sperm cells compared to males that did not sire outside of their social nest. In contrast, males that successfully defended all paternity within their social nest tended to have shorter heads and larger flagellum:head ratios compared to males that lost paternity in their social nest. While these patterns suggest that different sperm traits may be linked to success in different components of reproductive success, they should be interpreted with caution given the exploratory nature of this study and limited sample size, and further investigation is warranted.

## Introduction

Male reproductive success is typically determined by multiple factors beyond successful courtship and copulation with a female, including post-copulatory sexual selection. In most breeding systems, females copulate with more than one male, resulting in millions of sperm competing in a female’s reproductive tract to achieve fertilization [1]. Post-copulatory sexual selection is more pronounced in polygamous mating systems where females regularly mate with multiple males. However, even socially monogamous mating systems often include promiscuity. In birds, about 90% of species form seemingly monogamous social pairs for breeding, yet genetic polyandry occurs in roughly 76% of these species, indicating that extra-pair reproduction is common (especially among passerines) [2]. Therefore, despite their apparent monogamy, some males in these species likely still experience substantial sperm competition.

Importantly, the context of copulation may influence both the risk and intensity of post-copulatory sexual selection. Risk refers to the likelihood that sperm competition occurs at all, while intensity describes the degree of competition among ejaculates when sperm from multiple males are present. Extra-pair copulations generally involve both high risk and high intensity of post-copulatory sexual selection, as females that engage in extra-pair mating are more likely to have copulated with both their social mate and at least one extra-pair male. In contrast, within-pair copulations vary in risk depending on whether the female has mated with any other males. Additionally, females typically copulate more frequently with their social partner than with extra-pair males, often resulting in a greater quantity of the within-pair male’s sperm in the reproductive tract [3–5]. These differences in risk and intensity across mating contexts may favor distinct male behaviors and sperm morphologies.

**Fig 1:**
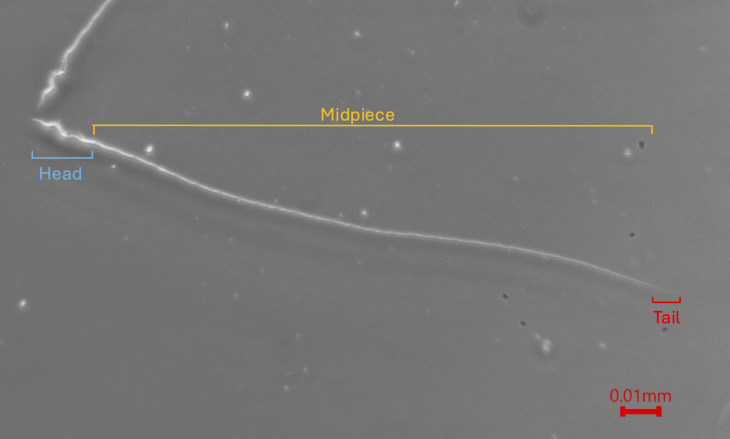
Mountain white-crowned sparrow sperm cell viewed at 40x using phase-contrast microscopy.

Multiple factors may influence whether a male’s sperm succeeds in fertilization. The occurrence of extra-pair paternity across many socially monogamous species [6], despite frequent copulations by within-pair males, suggests that fertilization success is not determined solely by the quantity of sperm in the female reproductive tract, as proposed by “the raffle principle” hypothesis [7,8]. Although within-pair males often copulate repeatedly and are assumed to transfer more sperm overall, extra-pair males may rival this through strategic allocation or precise timing of copulation. Other potential contributing factors include cryptic female choice and sperm morphology. Variation in sperm morphology can influence swimming speed, longevity, or the ability to navigate the female reproductive tract, all of which may affect fertilization success under post-copulatory selection [8,9]. In this study, we explore potential associations between sperm morphology and success in achieving extra-pair paternity and/or defending within-pair paternity. Sperm cells have three components, the head (which contains genetic information and the mechanism that allows for penetration of the egg), the midpiece (the location of the mitochondrial helix) and the tail (which facilitates sperm motility) (Fig 1) [10]. The flagellum, composed of the midpiece and tail, is often treated as a functional unit that facilitates sperm motility. The length of the head and flagellum, as well as derived traits such as total sperm length and the flagellum:head ratio, can vary among sperm cells within and/or among individuals and influence sperm motility and fertilization success [11–14].

Although strong post-copulatory sexual selection is expected to reduce variation in sperm morphology by favoring optimal traits [15] some socially monogamous species with high rates of extra-pair paternity still exhibit individual variation in sperm traits [16,17]. This variation may be shaped by sexual selection but could also result from genetic or environmental influences that contribute to phenotypic variation more generally. Differences in reproductive tactics may further influence how selection acts on sperm morphology across individuals. In socially monogamous systems, males can gain reproductive success either through within-pair paternity or by siring extra-pair offspring, and it is plausible that sperm morphology influences male success in one or both contexts. If these reproductive tactics are consistent over time or heritable, and if selection on sperm traits differ between tactics, they may help maintain variation in sperm traits across generations [18]. Some species with discrete alternative reproductive tactics, such as those that produce different morphological phenotypes with a corresponding suite of unique behaviors, often exhibit differences in sperm morphology and ejaculate traits among tactics [19,20], although many others do not [21,22]. Therefore, when assessing relationships between male traits and reproductive success, it is important to consider the context in which that success occurs (e.g., distinguishing between gaining extra-pair paternity versus maintaining within-pair paternity) rather than relying solely on total reproductive success.

Many studies have examined pre-copulatory male traits in relation to extra-pair paternity success with mixed results. For example, traits such as male age, song complexity, song length, and plumage ornamentation have sometimes been associated with increased extra-pair paternity success [23–27], though these correlations are often not strong or consistent correlation across studies [28]. For post-copulatory traits, previous studies that examined sperm morphology within individual components of reproductive success in social monogamy have also yielded conflicting results (S1 Table in Supporting Information) [11,16,29–33]. For example, in both bluethroats (*Luscinia svecica*; 25% of broods with extra-pair paternity) and house wrens (*Troglodytes aedon;* 37.6% of broods with extra-pair paternity), no relationships were found between morphological sperm traits in any reproductive context (total reproductive success, within-pair loss, or extra-pair success) [29,31]. However, the superb fairywren (*Malurus cyaneus;* 77–95% of broods with extra-pair paternity) the flagellum:head ratio was correlated with reproductive context and in opposite directions depending on mating tactic [16]. That is, males with high extra-pair success had sperm with longer heads and shorter tails and males that defended within-pair paternity had sperm with shorter heads and longer tails [16]. Fairywrens have some of the highest post-copulatory sexual selection of any passerine [17,34], and the maintenance of differences among males in sperm morphology indicates possible selection for males to pursue different alternative mating tactics [16].

In this study, we examine whether differences in sperm morphology are associated with a male’s likelihood of siring offspring within versus outside of his social nest in the mountain white-crowned sparrow (*Zonotrichia leucophrys oriantha;* MWCS), a species with moderate rates of extra-pair paternity (~40% of broods contain ≥1 extra-pair young) [35]. Rather than focusing solely on total reproductive output, we separately assessed two components of reproductive success: extra-pair success (siring offspring outside of the pair bond) and within-pair paternity loss (failing to sire offspring in one’s own nest). We tested two alternative hypotheses regarding the potential role of sperm morphology in these outcomes. First, we posit a *Context-dependent Selection Hypothesis*, which proposes that different sperm traits may confer advantages in different reproductive contexts (such as within-pair versus extra-pair fertilizations) potentially due to variation in female physiology, timing of copulations, or the number of competing ejaculates. Under this hypothesis, we expect that the sperm traits associated with success in gaining extra-pair paternity will differ from those associated with retaining within-pair paternity. Alternatively, we posit a *“One Sperm-size Fits All” Hypothesis* which suggests that there is a single optimal sperm morphology that enhances fertilization success across both contexts. This hypothesis predicts the same sperm traits will be associated with both higher extra-pair and lower within-pair paternity loss. Importantly, we acknowledge that reproductive outcomes such as extra-pair paternity and within-pair loss may also be influenced by behavioral factors like mate guarding or pursuit of extra-pair copulations. Our approach tests whether sperm morphology correlates with these outcomes, recognizing that any associations observed may reflect a combination of behavioral and post-copulatory mechanisms.

Given the modest sample size, we frame this as an exploratory study that tests specific hypotheses but interprets results as preliminary, offering insights and directions for future research rather than definitive conclusions. To test these hypotheses, we collected sperm from MWCS males during the 2022 breeding season and measured two primary morphological traits: head length and flagellum length (midpiece + tail). Because sperm morphology is repeatable within individuals at a given time point [12,36–38], a single ejaculate provides a representative snapshot of each male’s sperm phenotype during the breeding season. We also calculated two derived traits: total sperm length (head + midpiece + tail) and the flagellum:head ratio ((midpiece + tail)/ head) and we compare these four traits between males that did and did not sire at least one offspring outside of their pair bond (extra-pair success), and between males that protected paternity and sired all offspring in their social nest or lost paternity of at least one nestling in their social nest (within-pair loss). In addition, we examined whether sperm morphology was predictive of total male reproductive success (within-pair young + extra-pair young).

## Methods

### General field methods and study site

This field work was conducted in Tioga Meadows from May – July 2022. Tioga Meadows sits at ~3030m elevation in Mono County, CA, and is dominated by low willows and small stands of lodgepole pines (*Pinus contorta*). All procedures used on animals were approved by the Institutional Animal Care and Use Committee at UC Davis (Protocol #22827) and all relevant permits obtained prior to the onset of the study. Birds were captured via seed-baited Potter traps and banded with unique color bands and a USGS identifier band. Upon capture of adult birds, we collected a blood sample from the brachial vein for genetic information. We collected sperm from adult males (n = 18) via cloacal massage upon first capture, by gently applying pressure to the cloacal protuberance and collecting sperm with a capillary tube [37]. This is a rapid, minimally invasive technique that does not require anesthesia or analgesia [39]. To minimize discomfort, birds were handled for only a few minutes during sampling and released immediately afterward.

We fixed sperm samples in 5% formalin, 95% phosphate-buffered saline and applied parafilm to prevent evaporation; samples were stored at room temperature. All males were sampled between May 25^th^ and June 16^th^, except for one male captured on July 13^th^. Nests were located and monitored every 3 days; we collected blood samples from nestlings (via brachial vein) at 6 days post-hatching for genetic analysis. Blood samples of adults and offspring were blotted on Whatman FTA Elute© cards, dried, and stored at ambient temperature. For all males in the study, we only examined paternity at the first nest of the season to survive to fledging. We excluded failed nests and any subsequent nests (e.g., re-nests after a successful clutch) to avoid potential confounding effects of prior reproductive success on later reproductive decisions. Previous studies in other songbird species suggest that the outcome of an initial nesting attempt can influence the likelihood of extra-pair paternity in subsequent nests [40].

The social sire of each nest was determined through behavioral observations of color-banded males provisioning offspring. With this method, we can confidently assess whether a focal male lost paternity at his social nest. However, identifying whether a focal male sired extra-pair offspring in nests beyond our focal area is more challenging. To address this, we collected blood samples from nestlings and adults in a surrounding “buffer zone” beyond the core study site. Although we did not locate social nests for some males in this buffer zone, we could still detect if a focal male gained EPP by genetically matching him to offspring in sampled nests. Two such buffer-zone males were included in our EPP analyses after we confirmed they had sired offspring in focal nests on the core study area. Conversely, buffer-zone males not linked to any offspring within the sampled nests were excluded from analyses, as we could not confidently categorize them as having no EPP without broader sampling coverage.

### Sperm analysis

We used a P20 micropipette to slowly eject 10 µl of sperm/formalin/PBS samples in 4–5 thin rows along a glass microscope slide [15]. This was allowed to air dry for 3 hours. Slides were rinsed with deionized H_2_O to remove formalin crystals and air dried. The subsequent slide had sperm permanently fixed to the glass. One observer (C.H.) viewed sperm at 40x using a phase-contrast microscope and selected 10 sperm cells per male for imaging using an Amscope 500mp Camera. The 10 cells imaged were the first 10 in which all three sperm components (head, midpiece, tail) were clearly visible. Images were then randomized with blinded labels and subsequently measured in Image J by a single observer (C.H.). To assess measurement repeatability, we re-measured 1 sperm cell from each of 18 males for both head and flagellum length. Intra-class correlation coefficients (ICC) showed good repeatability for both traits (head ICC = 0.88; flagellum ICC = 0.92).

### Genetic analysis

DNA was extracted by eluting blood from Whatman FTA Elute Cards©; 3 mm punches of blood cards were put into 96-well plates, washed three times with 50 µL sterile H_2_O. After washing, we added 30 µL sterile H_2_O and incubated in a thermocycler at 95°C for 30 minutes. Following incubation, we vortexed for 1 minute, centrifuged the plates to draw the card punch to the bottom, and then aliquoted H_2_O into new plates (now containing the purified DNA) and stored at −20°C until ready for use. We quantified a subset of DNA samples using a Qubit to determine DNA concentrations for subsequent PCR amplification and discovered that nestling blood produced DNA concentrations 10x higher than adult blood, so nestling DNA was diluted by a factor of 10 for PCR amplification.

We then identified 8 microsatellite loci for PCR amplification to determine the parentage of nestlings that were sired by each male in the study (S2 Table). We ran these in two separate multiplexes, one with 5 microsatellites identified from previous work on the Puget Sound subspecies of white-crowned sparrow (*Zonotrichia leucophrys pugetensis)* and the other 3 microsatellites developed for use in other Emberizid species that have previously been used for *Z. l. oriantha* parentage assignments [35,41]. We prepared our multiplexes using 2x Qiagen MasterMix and fluorescently tagged our forward-labeled primers with DS-33 GeneScan Installation Standard dyes. Thermal cycling was accomplished with an Eppendorf Mastercycler Nexus Gradient; the protocol included an initial denaturation at 95°C for five minutes, followed by 35 cycles of 95°C for 15 seconds, annealing at 59°C (for the 5 *Z.l*. *pugetensis* primers) or 50°C (for the 3 Emberezid primers) for 15 seconds, and extension at 72°C for 30 seconds, followed by a final elongation step at 72°C for 10 minutes. To prepare our PCR products for fragment analysis, we added LIZ500 ladder and denatured at 95°C for five minutes. We sent PCR product to UC Berkeley DNA Sequencing facility for capillary electrophoresis on an Applied Biosystems Genetic Analyzer^TM^.

We estimated allele frequencies, null allele frequencies, and exclusion probabilities in Cervus 3.07 using genotypes from 365 individuals across 8 microsatellite loci. The mean number of alleles per locus was 12.6, mean expected heterozygosity was 0.75, and mean PIC was 0.72. The combined exclusion probability across all loci was 0.9917 with one parent known. Estimated null allele frequencies were low (< 0.05) for most loci, except for Zole_C12 (0.076, S2 Table) [42]. Parentage was determined by overlapping loci with potential males after loci from the female were accounted for. Candidate fathers were restricted to males present on the field site during the year of offspring conception. We allowed for up to 1 mismatching locus, 2 or more and the male was determined to not be the sire.

### Statistical analysis

We were interested in whether sperm morphology varies according to a male’s success at siring offspring within or outside of his social nest. Thus, we ran binomial generalized linear models to determine how sperm trait (a continuous predictor) increased the odds of a male siring an offspring within each reproductive component (a binary response). We coded extra-pair success as a binomial categorical variable, with males with no detected extra-pair offspring as “0” (n = 12), and males with 1 + extra-pair offspring as “1” (n = 6). Similarly, we coded within-pair loss as males that did not lose paternity within their nest as “0” (n = 8) and males that lost paternity in 1 + offspring within their nest as “1” (n = 8). For total reproductive success, we ran a linear model as it was a linear continuous response (total number of offspring produced per male within the breeding season). Specifically, predictor variables for all models included sperm component traits (head length and flagellum length) and derived traits (total length and flagellum:head ratio) averaged for each male. Because the sets of males contributing to extra-pair success and within-pair loss partially differed (e.g., some males did not have a social nest located), we analyzed these reproductive contexts in separate models rather than combining them with a context interaction term. We also assessed the relationship between head length and flagellum length by calculating Pearson’s correlation coefficient using the mean values per male.

We ran a total of eight binomial generalized linear models: four models (each with a single sperm component or derived trait, averaged per male) for each of the two binary response variables (extra-pair success and within-pair loss). This approach avoids overparameterizing the model and allows us to assess the relationship between each trait and each outcome separately. We assessed model fit and assumptions using residual plots. We also attempted a weighted model following the unbiased approach recommended by Cramer (2021), which included only the six males that sired at least one extra-pair offspring [43]. Because of the small sample size and convergence issues, this model was used as a supplementary analysis to assess robustness rather than a primary test. Additionally, we ran four standard linear models (1 for each sperm trait) with total seasonal reproductive success as the predictor (n = 16). For all analyses, we used the mean value of each sperm trait per male, rather than repeated measurements, to avoid pseudoreplication because each male had only one reproductive outcome per context. All models were run using base R functions and p-values calculated using the Satterthwaite method using “lmerTest” in R [44,45].

## Results

### Male fertilization success

In the Tioga MWCS population for 2022, 52.6% (10/19) of broods had at least one extra-pair young, and 25.3% of young (19/75) were sired by EP males. Two males included in this dataset experienced total loss of within-pair paternity and no additional extra-pair paternity. Both of these fully cuckolded males produced sperm that were within the normal range of morphological variation observed in our sample, suggesting their lack of paternity was not due to obvious sperm abnormalities, in contrast to findings in other species where some fully cuckolded males produced no sperm [46].

### Sperm component traits

**Fig 2:**
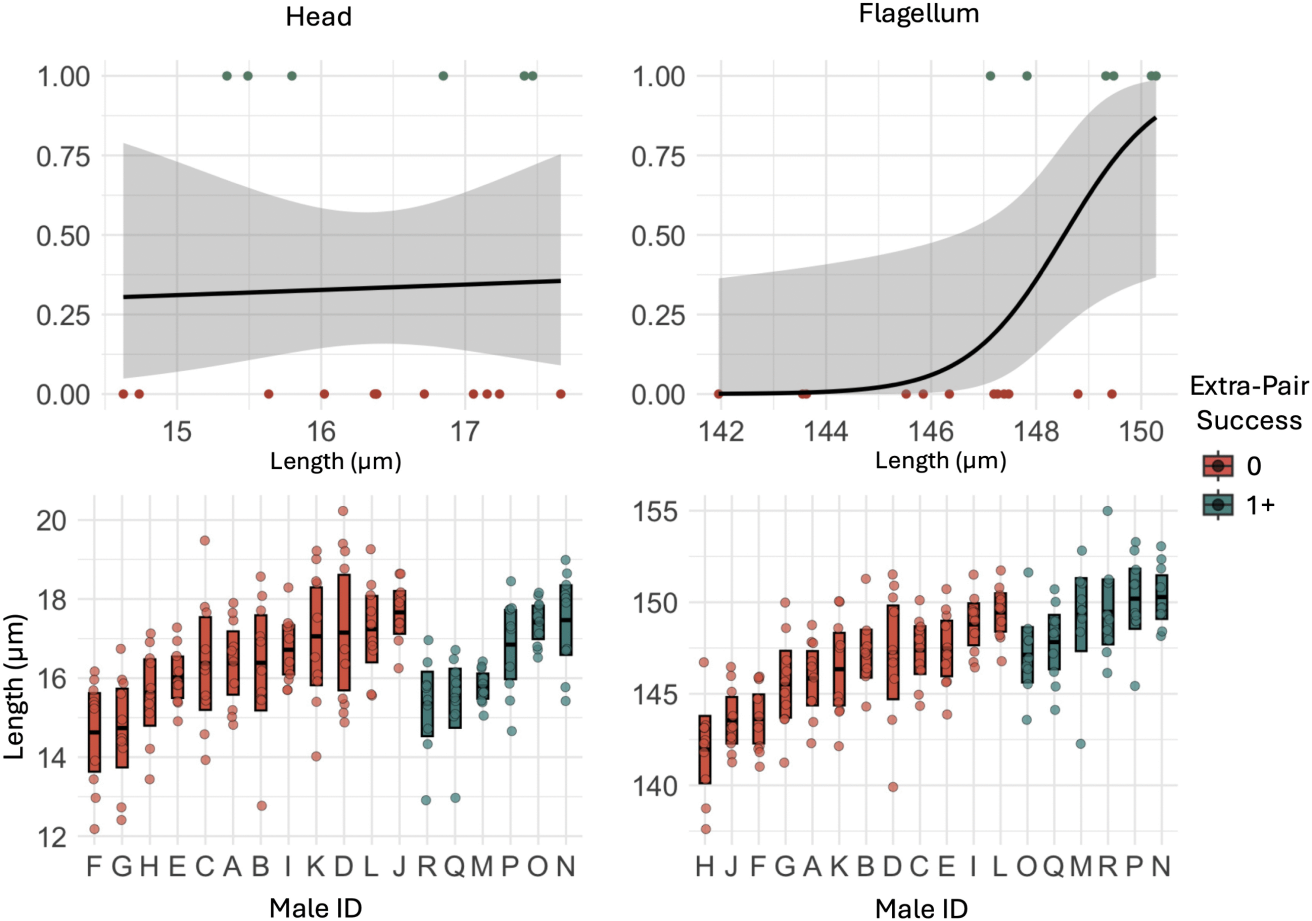
Extra-pair success and sperm trait lengths (µm) of Mountain white-crowned sparrows captured during the breeding season (May – June 2022) at Tioga Pass, California. The top row of figures indicates generalized linear model results, and the lower panels show raw data per male: individual points represent all sperm cells measured (10 per male), with group means ± 95% confidence intervals.

There was no significant relationship between component part sperm traits and total seasonal reproductive success (Table 1). However, we found that sperm component traits predicted a male’s ability to gain paternity outside of his nest: males that achieved at least a single extra-pair offspring had longer flagella than those that did not sire young outside of their nest (p = 0.04; Fig 2; Table 1). We found no significant difference in the lengths of the head or tail components of sperm between these two groups of males. Additionally, we detected a weak positive correlation between head length and flagellum length (r = 0.28, n = 18). We also ran models using a weighted approach for extra-pair success, which produced qualitatively similar results to those reported here.

**Table 1:** Binomial generalized linear model results examining the relationship between sperm traits (head length, flagellum length, total length, and flagellum:head ratio) as predictors and reproductive outcomes as binomial response variables: extra-pair success (0 = no extra-pair offspring, 1 = ≥1 extra-pair offspring) and within-pair loss (0 = no paternity loss, 1 = ≥ offspring lost). Total reproductive success (continuous) was analyzed using linear models. Each row represents a separate model.

Extra-pair Success Binomial Generalized Models				
	Estimate	SE	z value	Pr (> |z|)
Head	0.076	0.557	0.14 (1, 16)	0.89
Flagellum	1.087	0.523	2.08 (1, 16)	0.04
Total length	0.702	0.387	1.815 (1, 16)	0.07
Flagellum:head ratio	0.5974	1.042	0.573 (1, 16)	0.57
**Within-pair Loss Binomial Generalized Models**				
	**Estimate**	**SE**	**z value**	**Pr (> |z|)**
Head	1.325	0.741	1.79 (1, 14)	0.07
Flagellum	−0.152	0.226	−0.68 (1, 14)	0.5
Total length	0.016	0.189	0.087 (1, 14)	0.93
Flagellum:head ratio	−2.776	1.495	−1.856 (1, 14)	0.06
**Total Seasonal Reproductive Success Linear Models**				
	**Estimate**	**SE**	**t value**	**Pr (> |z|)**
Head	−0.476	0.731	−0.65 (1, 15)	0.52
Flagellum	0.383	0.277	1.39 (1, 15)	0.19
Total length	0.233	0.248	0.940 (1, 15)	0.36
Flagellum:head ratio	1.339	1.302	1.028 (1, 15)	0.32

**Fig 3:**
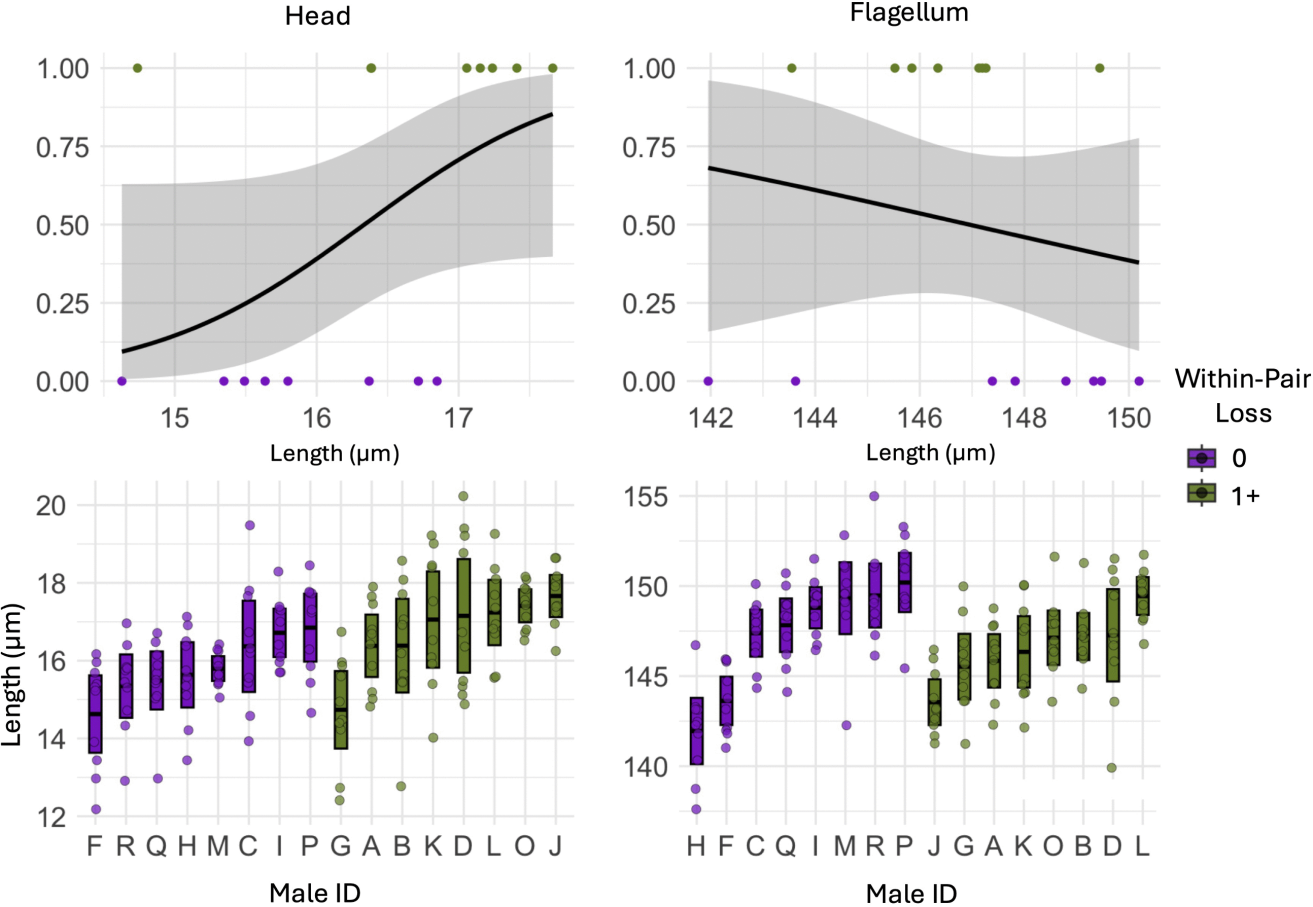
Within-pair loss and sperm trait lengths (µm) of Mountain white-crowned sparrows captured during the breeding season (May – June 2022) at Tioga Pass, California. The top row of figures indicates generalized linear model results, and the and the lower panels show raw data per male: individual points represent all sperm cells measured (10 per male), with group means ± 95% confidence intervals.

We found weak evidence that males who maintained complete paternity within their social nest had smaller heads compared to males that lost paternity in one or more offspring in their social nest (p = 0.07; Fig 3; Table 1). We found no significant difference in flagellum length between the two groups of males.

### Derived sperm traits

**Fig 4:**
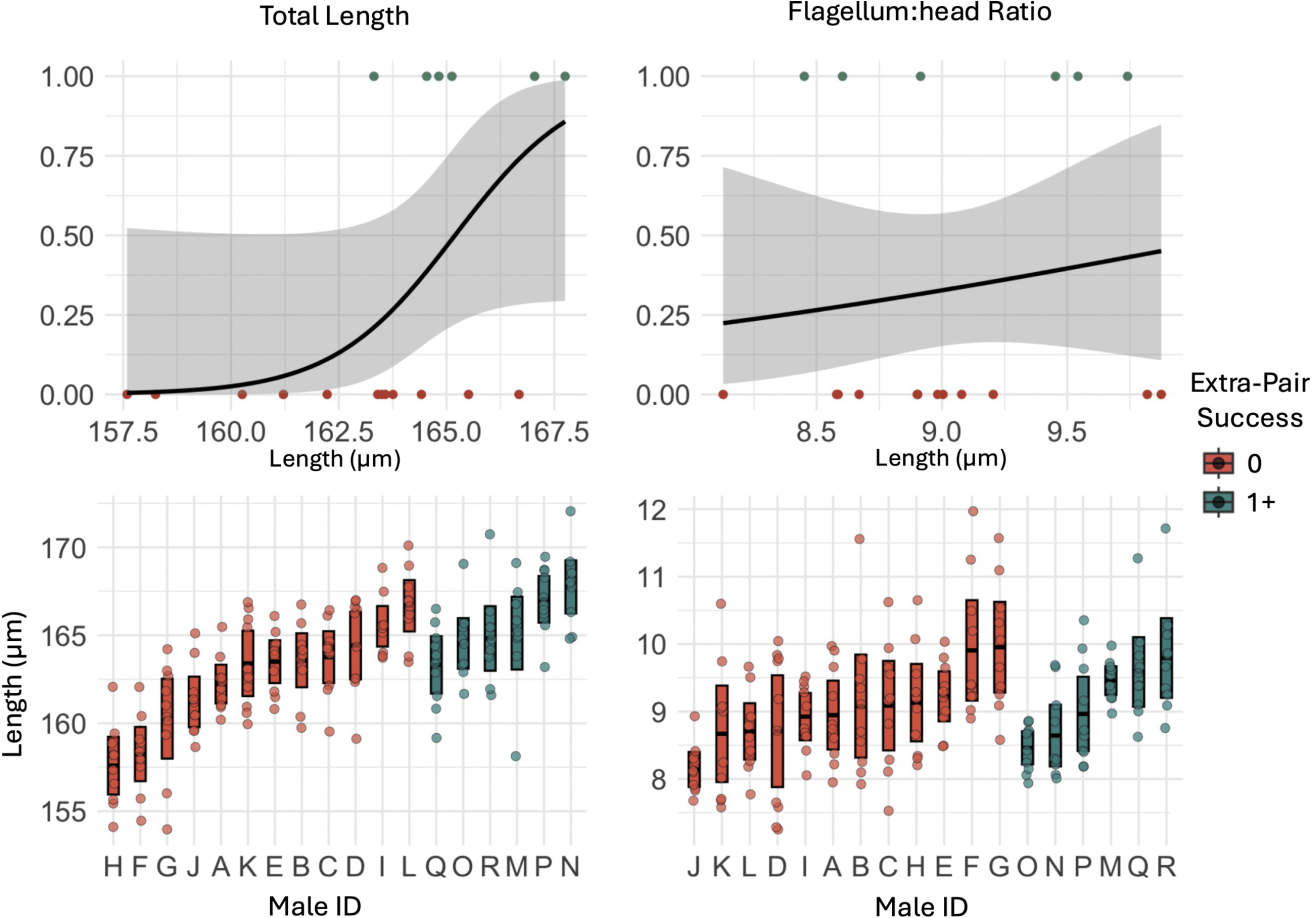
Extra-pair success and derived sperm traits of mountain white-crowned sparrows captured during the breeding season (May – June 2022) at Tioga Pass, California. The top row of figures indicates generalized linear model results, and the and the lower panels show raw data per male: individual points represent all sperm cells measured (10 per male), with group means ± 95% confidence intervals.

There was no significant relationship between derived sperm traits and total seasonal reproductive success (Table 1). However, we found weak evidence that males who sired at least one extra-pair offspring had longer total length of sperm compared to males who did not sire young outside of their social nest (p = 0.0695; Fig 4; Table 1). We found no significant difference in flagellum:head ratio between the two groups of males.

**Fig 5:**
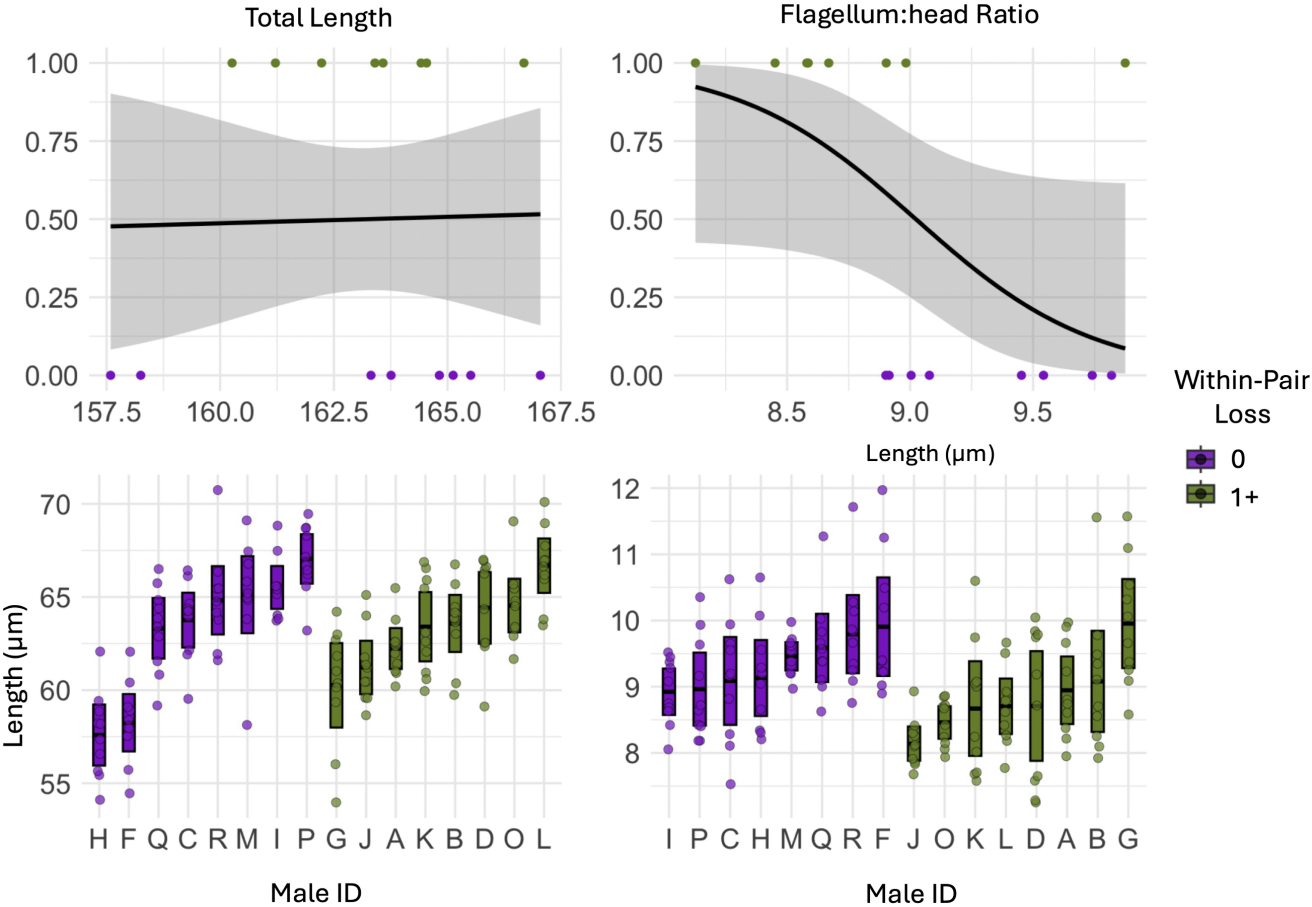
Within-pair loss and derived sperm traits of Mountain white-crowned sparrows captured during the breeding season (May – June 2022) at Tioga Pass, California. The top row of figures indicates generalized linear model results, and the lower panels show raw data per male: individual points represent all sperm cells measured (10 per male), with group means ± 95% confidence intervals.

We found weak evidence that males who maintained complete paternity within their social nest had higher flagellum:head ratios compared to males who lost paternity in in one or more offspring within their social nest (p = 0.063; Fig 5; Table 1). We found no significant difference in the sperm total length between the two groups of males.

## Discussion

By examining components of reproductive success (extra-pair versus within-pair) separately we revealed potential relationships between fertilization success and sperm morphology. We found that males who achieved extra-pair success had significantly longer flagella compared to males who did not. Additionally, we observed weak evidence suggesting that males who achieved extra-pair success had longer total sperm lengths compared to males who did not. For within-pair paternity, we found weak evidence that males who retained full paternity at their social nest had smaller heads and larger flagellum:head ratios than males who lost paternity. These results indicate that different aspects of sperm morphology are advantageous in achieving extra-pair versus within-pair reproductive success, which tentatively supports our *Context-Dependent Sperm Hypothesis*. We did not find a tradeoff between these morphologies (i.e., we did not observe negative effects on success in one reproductive component and positive effects in the other) and therefore do not have evidence that success in one tactic comes at the cost of success in the other. Due to sample size limitations and resulting statistical power constraints, our results should be interpreted cautiously as exploratory. While we observe suggestive patterns consistent with the *Context-Dependent Sperm Hypothesis,* further studies with larger samples are needed to confirm these findings.

Because males that achieved extra-pair success had longer flagella than unsuccessful males, we had also expected to see higher flagellum:head ratios in these successful versus unsuccessful males; however, we did not detect a significant difference (p = 0.57). One possible explanation for the lack of a relationship between flagellum:head ratio and extra-pair success (despite considerably larger flagella in successful males) is that three males in the extra-pair success group also had above-average head lengths (see S1 Fig in Supporting Information) which may have swamped any effect of larger flagella (see Supporting Information for S1 Fig). Similarly, despite weak evidence for smaller heads in males that preserved paternity at the social nest, we did not find evidence for significant differences in total length among males that lost versus preserved paternity. This may be explained by the relatively small percentage that the head makes up of the total sperm cell length (~10%) compared to midpieces (~87%).

In this study, we found that both flagellum and total length were higher in males successful in achieving extra-pair paternity versus those who are unsuccessful, which is consistent with existing literature on the correlation between sperm size and fertilization success under highly competitive environments. In captive zebra finches (*Taeniopygia guttata*), an experiment comparing sperm cells from ejaculates to those that reached the ovum (as determined by dissecting the pre-vitelline layers of fertilized eggs) revealed that longer sperm were more successful at reaching and fertilizing the ova [13]. The flagellum may play a key role in sperm motility, potentially influencing swimming performance and fertilization success. Overall, these patterns suggest a complex relationship between sperm morphology and components of reproductive success that warrants further investigation.

Theoretically, longer flagella should improve swimming speed, whereas a larger head may increase drag [47]. However, empirical studies in birds provide mixed support for this relationship. In house sparrows (*Passer domesticus*), one study found that smaller heads were associated with faster swimming sperm [48], but subsequent work in the same species reported different or weaker patterns [12,49]. Reviews have also questioned whether the flagellum:head ratio is a reliable hydrodynamic index for avian sperm swimming [43,49]. In our study, males that successfully defended paternity at their social nest tended to have smaller heads and higher flagellum:head ratios compared to males that lost paternity, which could reflect a role for faster swimming sperm, but given the conflicting evidence across studies, we interpret this finding cautiously.

There are important caveats and limitations in the results presented here. This observational field study was motivated by the question of whether a male’s reproductive tactic was predictive of his sperm morphology. But to quantify male tactics, we would need to observe male effort in pursuing extra-pair tactics (e.g., courtship and copulations with extra-pair females) or within-pair tactics (e.g., mate guarding), whereas we were limited to examining reproductive outcomes (success or lack of success in these different contexts). It is possible that a male invested heavily in extra-pair tactics and did not achieve any extra-pair copulations, or that a male achieved extra-pair copulations but did not fertilize any extra-pair young due to post-copulatory sexual selection. Further studies are needed to directly link sperm morphology to observable reproductive tactics. Additionally, sperm morphology was measured at a single time point per male, which provided a representative snapshot of each male’s phenotype during the breeding season. Repeated sampling in future studies would allow for tests how stable sperm traits are over time, and how that stability relates to fertilization success.

Further, we only reported paternity for first nests that survived to fledging success, but some males in this study could have had previous nests that were lost to predators or weather before fledging and/or additional reproductive success in later nests that were not sampled. Studies on Japanese great tits (*Parus minor*) indicate that early nest failures can increase female propensity to pursue extra-pair mates [40,50]. Indeed, one of our focal males (Male E) had three nests lost to predation over the course of the breeding season and thus was excluded from our within-pair analysis due to no within-pair nests that survived to fledging.

Additionally, while we have confidence in our within-pair loss dataset, our extra-pair success dataset may not be as robust in the case that we did not detect a male’s extra-pair offspring (e.g., focal male sired offspring in a nest not sampled within our field site). To mitigate this risk, we sampled nests in a “buffer zone” surrounding the focal field site and we also sampled adult males in this buffer zone in case they sired offspring within the focal site. Previous work on this field site reveals that most extra-pair sires often have their social nests in close neighboring vicinities as those they achieved extra-pair paternity in (*Hawkins, unpublished data*), and thus we feel the buffer zone provides increased confidence that extra-pair offspring would not go undetected.

We acknowledge that in many songbirds, sperm traits can vary with date [31,51]. This study is based on a relatively small sample size, which limits the number of covariates (e.g., sampling date) we could include in our models without substantially reducing statistical power. Most males were sampled within a relatively short window (May 25^th^ – June 16^th^, 2022), one male (Male K) was captured later (July 13^th^, 2022). Since his sperm traits did not noticeably differ from other males, we retained him in the analysis to avoid unnecessarily reducing our dataset. Future research with larger datasets and repeated sampling throughout the breeding season should aim to account for potential temporal variation to fully understand how sperm morphology may influence components of reproductive success.

Although some sperm components may co-vary due to shared developmental or functional constraints, we found only a weak correlation between head length and flagellum length in this dataset (r = 0.28), which supports our treatment of these traits as independent variables in our models. Nonetheless, the limited sample size means we cannot rule out subtle relationships between traits that may affect interpretation of their individual contributions to reproductive success in either context. Additionally, we were unable to test for an interaction between components of reproductive success (within-pair vs. extra-pair) and sperm traits because not all males included in the extra-pair success dataset had corresponding within-pair data, largely due to some males nesting outside the monitored field site (e.g., in the “buffer zone”).

Finally, not much is known in MWCS about the time of day that extra-pair copulations occur, and it is possible that our capture effort itself could leave the female unguarded and available for an extra-pair copulation while we capture and process the social male, thus affecting within-pair loss outcomes, so we did our best to process and release males as fast as possible while maintaining safety procedures. We have good reason to believe our capture effort did not impact extra-pair paternity rates, as previous work on this population involved rigorous methods to reduce time in trap (males were watched continuously from a distance and removed from traps immediately upon capture during the female lay period), during which population EPP rates ranged from 44–68% over a three-year period [35]; our 52.6% EPP rate falls well within this range.

Our results inspire multiple avenues for further research. For example, additional studies are needed to test possible mechanisms underlying the results observed here, with differential success of different sperm morphologies in different reproductive contexts. Further research is also needed to address other aspects of morphology that may affect the outcome of post-copulatory sexual selection, such as the helical keel of the head, which may be related to swimming speed [52]. Additionally, ejaculate characteristics like sperm count and ejaculate volume, which require more controlled conditions during sperm sampling, may play an important role in post-copulatory sexual selection. Male red junglefowl (*Gallus gallus*), for example, adjust ejaculate attributes dependent on female novelty, attractiveness, and the presence of other males [53–55].

Another area requiring further research is the role of females in determining the success of different behavioral tactics in males and the outcome of post-copulatory sexual selection. Females are not passive participants in the behavioral dynamics of extra-pair/within-pair reproductive tactics and can either choose to pursue or avoid extra-pair copulations. Females may exercise cryptic mate choice by altering timing and number of copulations, as well as the vaginal environment in which sperm competition occurs [56]. Across avian taxa, variation in female sperm storage organs and population promiscuity levels are significant drivers of sperm length evolution; high promiscuity rates can often lead to stabilizing selection for optimal sperm morphology, whereas high variation among females in female sperm storage may cause disruptive selection by favoring long and short sperm length [57]. Directly testing for cryptic female choice, particularly in field environments, remains logistically challenging [58] but it is required for a complete understanding of sperm competition and male reproductive tactics in any system.

## Conclusions

In this study, we found consistent support for the Context-Dependent Sperm hypothesis where the relationship between fertilization and component trait or derived measures of sperm morphology differed in within-pair versus extra-pair matings. None of these morphological traits were under conflicting selection, with the same trait under positive selection in one context and a negative selection in the other. Therefore, our results do not reveal a trade-off between pursuing one tactic over another. We also never found a trait that was correlated in consistent directions across both contexts, suggesting that there is no single morphology best for increasing total reproductive success. Our results demonstrate the importance of specifying the context of reproductive success when studying sexual selection in mating systems in which reproductive investment can occur within and/or outside of one’s social nest (e.g., social monogamy, social polyandry), as well as the importance of examining selection on different components of sperm morphology separately. Although models using total reproductive success can capture overall selection patterns, examining distinct reproductive components can reveal biologically meaningful variation that may otherwise be obscured.

## Supporting information

S1 Table A synthesis of all current papers that examined relationships between sperm morphology within specific reproductive contexts. Four papers did not find evidence for different morphologies across reproductive context, but one of the three (Laskemoen et al 2010) found a positive relationship between Midpiece:Total Length ratio with total reproductive success. Rowe et al 2022 found a weak association between lower flagellum:head ratio and higher within-pair success, although confidence intervals overlapped zero and they largely concluded no associations between sperm morphological traits and reproductive contexts. Calhim et al 2011 found a negative relationship with flagellum:head ratio and extra-pair success and a positive relationship in within-pair loss, suggesting the tactics conflict with one another in superb fairywrens (*Malurus cyaneus*). Both Yang et al 2021 and the current paper’s results found a positive relationship between total length and extra-pair success.
(TIF)

S2 Table Microsatellite loci for parentage assignments. (TIF)

S3 Table Robustness of results to different methods of summarizing multiple sperm measurements per male. Each male had 10 sperm cells measured but only one reproductive outcome, so including Male ID as a random effect would artificially inflate degrees of freedom in our binomial models. In the main text (Table 1), we summarized measurements for each male using the mean value across the ten sperm cells. To verify that our results were not an artifact of this summarization method, we re-ran all analyses using two alternatives: (1) the first sperm cell measured for each male and (2) the median value across the 10 sperm cells. Results were consistent across all approaches.
(TIF)

S1 Fig Sperm components scaled relative to the average of the population for each component, sorted by extra-pair success. The vertical pink line represents the average length for that component. The total length column is also scaled relative to the average, but the individual components are accurate proportions to the total length of the cell.
(TIF)
